# 16S rRNA Metabarcoding Used to Identify the Etiology of Infective Endocarditis Agents

**DOI:** 10.17691/stm2026.18.1.04

**Published:** 2026-02-27

**Authors:** A.V. Sinitskaya, A.E. Kostunin, M.V. Khutornaya, A.O. Poddubnyak, O.N. Hryachkova, M.A. Asanov, A.E. Tupikin, M.R. Kabilov, A.N. Stasev, M.Y. Sinitsky

**Affiliations:** PhD, Senior Researcher, Laboratory of Genomic Medicine, Department of Experimental Medicine; Research Institute for Complex Issues of Cardiovascular Diseases, 6 Academician Barbarash Blvd., Kemerovo, 650002, Russia; PhD, Senior Researcher, Laboratory of New Biomaterials, Department of Experimental Medicine; Research Institute for Complex Issues of Cardiovascular Diseases, 6 Academician Barbarash Blvd., Kemerovo, 650002, Russia; Researcher, Laboratory of Genomic Medicine, Department of Experimental Medicine; Research Institute for Complex Issues of Cardiovascular Diseases, 6 Academician Barbarash Blvd., Kemerovo, 650002, Russia; Laboratory Research Assistant, Laboratory of Genomic Medicine, Department of Experimental Medicine; Research Institute for Complex Issues of Cardiovascular Diseases, 6 Academician Barbarash Blvd., Kemerovo, 650002, Russia; PhD, Researcher, Laboratory of Genomic Medicine, Department of Experimental Medicine; Research Institute for Complex Issues of Cardiovascular Diseases, 6 Academician Barbarash Blvd., Kemerovo, 650002, Russia; Junior Researcher, Laboratory of Genomic Medicine, Department of Experimental Medicine; Research Institute for Complex Issues of Cardiovascular Diseases, 6 Academician Barbarash Blvd., Kemerovo, 650002, Russia; Junior Researcher, Center for Collective Use “Genomics”; Institute of Chemical Biology and Fundamental Medicine, Siberian Branch of the Russian Academy of Sciences, 8 Lavrentuev Prospekt, Novosibirsk, 630090, Russia; PhD, Head of the Center for Collective Use “Genomics” Institute of Chemical Biology and Fundamental Medicine, Siberian Branch of the Russian Academy of Sciences, 8 Lavrentuev Prospekt, Novosibirsk, 630090, Russia; MD, PhD, Senior Researcher, Laboratory of Heart Defects, Department of Heart and Vascular Surgery; Research Institute for Complex Issues of Cardiovascular Diseases, 6 Academician Barbarash Blvd., Kemerovo, 650002, Russia; PhD, Head of the Laboratory of Genomic Medicine, Department of Experimental Medicine; Research Institute for Complex Issues of Cardiovascular Diseases, 6 Academician Barbarash Blvd., Kemerovo, 650002, Russia

**Keywords:** infective endocarditis, bacterial agents, 16S metabarcoding, native cardiac valves

## Abstract

**The aim of the study** was to estimate the efficiency of identifying bacterial agents in native cardiac valves affected by infective endocarditis using 16S rRNA metabarcoding.

**Materials and Methods:**

The study material involved 20 native cardiac valve samples from 16 patients. Sequencing was carried out in the Center for Collective Use “Genomics” (Institute of Chemical Biology and Fundamental Medicine, Siberian Branch of the Russian Academy of Sciences, Russia) on a sequenator MiSeq (Illumina, USA) using MiSeq Reagent Kit v3 (2×300 bp; Illumina, USA).

**Results:**

The study revealed major microorganisms (their portion in the sample under study was over 5% from all identified bacterial agents) belonging to *Streptococcus* (40% of all valves studied), *Sphingomonas* (35%), *Pseudomonas* (35%), *Roseateles* (25%), *Phyllobacterium* (25%), and *Enterococcus* (15%). Moreover, the studied valves were found to have rare cases of *Ralstonia pickettii*, as well as *Bacillus* and *Klebsiella* representatives.

**Conclusion:**

The findings demonstrated the efficiency of 16S rRNA metabarcoding in identifying bacterial agents compared to routine noninvasive diagnostic methods. Native cardiac valves isolated from the patients with infective endocarditis were characterized by numerous opportunistic pathogenic bacteria in negative blood cultures.

## Introduction

Infective endocarditis (IE) is an inflammatory condition of primarily bacterial origin characterized by agents localized on native cardiac valval structures, endocardium, as well as on prosthetic cardiac valves and pacemakers [1]. Epidemiological studies have demonstrated an increase in IE incidence rate and the growth of fatal cases due to the complications related to the pathological condition [2, 3]. Agent identification is a crucially important aspect for IE diagnosis; it has an impact on antibiotic therapy and recurrent bacteremia risk in patients with cardiac valve prostheses [4]. The gold standard of identifying IE etiology is considered to be a microbiological blood culture or cardiac valval tissue excised. However, it should be noted that in most cases the cultures appear to be negative. In this situation, according to applicable clinical guidelines [5], it is possible to use molecular genetic methods (e.g., polymerase chain reaction) [6]. Preoperative antibiotic therapy administered in an inpatient department implies difficulties in detecting the agents using standard methods; therefore, there is an increasing demand for more accurate diagnosis and identification of bacterial agents in order to decrease IE mortality [7].

Currently, one of the promising methods is next-generation sequencing (NGS), which enables to identify pathogens in cases when standard molecular genetic tests yield no positive result [8]. It should be mentioned that the sensitivity and specificity of the method are higher for explanted tissues compared to peripheral blood [9]. At the initial stage of metagenomic 16S rRNA sequencing, there is the amplification of variable parts using specific primers followed by sequencing the resulted product that enables to identify a bacteriological agent, its genus, species. 16S rRNA gene, which encodes the 16S ribosome subunit, has been found in all bacteria, it includes highly conserved regions with variable sequences [10]. The unquestionable advantage of the method is detecting nonviable microorganisms by a bacterial DNA, as well as the bacterial agents in patients after antibiotic therapy [11].

**The aim of the study** was to estimate the efficiency of identifying bacterial agents in native cardiac valves affected by infective endocarditis using 16S rRNA metabarcoding.

## Material and Methods

### Study group

Study material involved 20 native cardiac valve specimens affected by IE, which were operatively taken from 16 cardiosurgical patients. It should be noted that the most cases were left-sided IE, while right-sided IE was found in 1 patient.

Clinical patient profiles:

male gender — 12 patients (75%);
mean age (Me [Q1; Q3]) — 57.0 [35.0; 63.0] years;
body mass index (mean±SD) — 24.63±3.95;
left ventricular ejection fraction (mean±SD) — (63.64±9.50)%;
pulmonary hypertension — 10 patients (62.5%);
hypertensive disease — 7 patients (43.75%);
chronic cardiac failure — 16 patients (100%);
atrial fibrillation — 3 patients (18.75%);
acute cerebrovascular disease — 4 patients (25%);
chronic renal failure — 8 patients (50%);
chronic obstructive pulmonary disease — 4 patients (25%);
antibiotics taken prior to admission — 12 patients (75%);
involved valve: aortal, mitral and tricuspid — 9 (45%) patients, 10 (50%) patients, and 1 (5%) patient, respectively.

Laboratory findings (Me [Q1; Q3]):

leukocytes —7.20 [5.40; 9.30]·10^9^/L;
neutrophils — 4.40 [2.90; 5.85]·10^9^/L;
C-reactive protein — 4.80 [2.00; 16.40] mg/L;
erythrocyte sedimentation rate — 18.0 [5.50; 51.50] mm/h.

The study was carried out in accordance with the standards of Good Clinical Practice and Declaration of Helsinki (2024). All participants signed the informed consent to be involved in the research. The present study was approved by a local Ethics Committee of Research Institute for Complex Issues of Cardiovascular Diseases (protocol No.1 dated January 26, 2024).

Infective endocarditis diagnosis was made based on clinical, microbiological, and laboratory data, and echocardiography (echoCG) findings. According to echoCG, all patients involved in the study, had movable vegetations of the valves. Additionally, the diagnosis was verified in accordance with Duke international criteria. A week prior to the surgery, the patients under study had a peripheral blood test for microbiological testing in three repeats, the interval being 6 h. The histological Gram staining was performed using a commercial kit (Abcam, Great Britain).

### 16S rRNA metabarcoding

DNA was isolated from the explanted valval specimens using HostZERO Microbial DNA Kit (Zymo Research, USA). V3–V4 region of 16S rRNA gene was amplified using the primers: 343F (5’-CTCCTACGGRRSGCAGCAG-3’) and 806R (5’-GGACTACNVGGGTWTCTAAT-3’) containing the adapter sequences (Illumina, USA), a linker and a barcode [12]. The amplification was performed in the reaction mixture, 20 μl (in three repetitions) containing Hot Start Taq DNA polymerase, 0.4 U (Biolabmix, Russia) and single HS Taq PCR-buffer (Biolabmix, Russia); 0.2 μmol of forward and reverse primers; DNA, 10 ng; MgCl_2_, 2.3 mM (Biolabmix, Russia), and dNTP, 0.2 mM (Life Technologies, USA). The number of cycles was selected for each DNA sample, performing PCR with fluorescent signal detection in a real-time mode on a DNA-amplifier CFX-96 (Bio-Rad, USA). As an intercalating fluorophore we used a dye — Eva488 (Lumiprobe, USA). A cycle selection criterion was a fluorescent signal located at a linear logarithmic growth phase. In case of low PCR efficiency, the maximal number of cycles was 34. The target product yield was analyzed on MultiNA using 12000 DNA Base Pair Kit (Shimadzu, Japan). PCR was performed as follows: 5 min at 95°C; then up to 40 cycles — 15 s at 95°C, 15 s at 62°C, 30 s at 72°C; the last stage — 5 min at 72°C. Amplicons (by 200 ng each) were united and cleaned using magnetic particles AMPure XP Beads (Beckman Coulter, USA) according to a manufacturer’s instruction.

Sequencing was carried out in the Center for Collective Use “Genomics” (Institute of Chemical Biology and Fundamental Medicine, Siberian Branch of the Russian Academy of Sciences) on a sequenator MiSeq (Illumina, USA) using MiSeq Reagent Kit v3 (2×300 bp; Illumina, USA). The number of reads obtained for each sample was determined using the program Seqkit [13]. The quality was studied using FastQC [14]. Pair sequences were analyzed using UPARSE-scripts [15] and Usearch v. 11.0.667 [16]. Bioinformation processing included the overlapping of paired reads, the filtration by quality and length, the consideration of similar sequences, dropping of singletons, deletion of chimeras, and obtaining OTU using a clustering algorithm UPARSE [17]. The taxonomic affiliation of OTU sequences was determined using SINTAX [18] and the reference base 16S RDP training set v. 19 [19].

### Statistical data processing

The data were statistically processed using the program Prism 8 (GraphPad Software, USA). Quantitative results were represented as arithmetic mean (M) and standard deviation (SD), as well as median (Me), lower and higher quartiles [Q1; Q3].

## Results

A microbiological blood test carried out in accordance with a standard protocol and described in clinical guidelines [5] enabled to state IE etiology in only 25% cases, when IE agents were gram-positive bacteria. Histological Gram staining of the incised cusps of native cardiac valves succeeded in identifying the bacteriological agents in 45% cases (see the Table). In most cases, 16S rRNA metabarcoding revealed major microorganisms (their portion in the samples under study was over 5% of all identified bacterial agents) belonging to *Streptococcus* (40% of all valves studied), *Sphingomonas* (35%), *Pseudomonas* (35%), *Roseateles* (25%), *Phyllobacterium* (25%), and *Enterococcus* (15%). However, for the most part of mitral valves and the small portion of aortal valves there was hardly the variety of bacteria, just one major genus being present: either *Streptococcus*, *Klebsiella* or *Enterococcus* (see the Figure).

**Table:** Taxonomic variety of bacteria revealed in patients with infective endocarditis (20 valves)

Valve type	Culture study	Gram staining findings	16S rRNA sequencing (percentage in a sample)
Aortal	Sterile culture	Negative	*Pseudomonas stutzeri* (27%) unc_*Acinetobacter* (14%) unc*_Roseateles* (9%) unc_*Pseudomonas* (8%) unc*_Sphingomonas* (6%)
Aortal	*Enterococcus faecalis*	Negative	unc_*Bacillus* (67%) unc_*Enterococcus* (8.3%)
Mitral	*Enterococcus faecalis*	Negative	unc_*Enterococcus* (100%)
Aortal	Sterile culture	Rods/cocci	*Ralstonia pickettii* (55%) *Pseudomonas stutzeri* (5.5%)
Mitral	Sterile culture	Negative	unc_*Roseateles* (15%) *Pseudomonas stutzeri* (11%) unc_*Acinetobacter* (6.7%) unc_*Reyranella* (6.1%)
Mitral	Sterile culture	Negative	*Pseudomonas stutzeri* (19%) *Sphingobium limneticum* (9.6%) unc*_Acinetobacter* (8.4%) unc*_Pseudomonas* (8.0%) unc*_Roseateles* (6.7%) unc*_Agrobacterium* (6.4%)
Aortal	*Streptococcus gordonii*	Cocci	*Pseudomonas stutzeri* (19%) unc*_Streptococcus* (13%) unc*_Agrobacterium* (10%) unc*_Pseudomonas* (6.3%) unc*_Roseateles* (5.8%) unc*_Sphingomonas* (5.5%)
Mitral	*Streptococcus gordonii*	Cocci	unc_*Streptococcus* (47%) *Pseudomonas stutzeri* (12%)
Tricuspid	Sterile culture	Negative	*Pseudomonas stutzeri* (16%) unc_*Roseateles* (15%) unc*_Sphingomonas* (11%) unc*_Acinetobacter* (13%) *Sphingobium limneticum* (9.7%)
Aortal	Sterile culture	Cocci	unc*_Streptococcus* (72%) unc*_Sphingomonas* (6.5%) unc*_Phyllobacterium* (5.2%)
Mitral	Sterile culture	Cocci	unc*_Streptococcus* (99%)
Mitral	Sterile culture	Negative	unc*_Sphingomonas* (17%) unc*_Phyllobacterium* (32%)
Aortal	Sterile culture	Cocci	unc_*Streptococcus* (99%)
Aortal	Sterile culture	Cocci	unc*_Klebsiella* (100%)
Mitral	Sterile culture	Negative	unc*_Streptococcus* (100%)
Aortal	*Enterococcus faecalis*	Cocci	unc_*Enterococcus* (100%)
Aortal	Sterile culture	Cocci	unc*_Streptococcus* (100%)
Mitral	Sterile culture	Cocci	unc*_Streptococcus* (86%)
Mitral	Sterile culture	Rods/cocci	unc*_Sphingomonas* (11%) unc*_Phyllobacterium* (26%)
Aortal	Sterile culture	Negative	unc*_Sphingomonas* (7.5%) unc*_Phyllobacterium* (30%)

N o t e: unc — unclassified.

**Figure:**
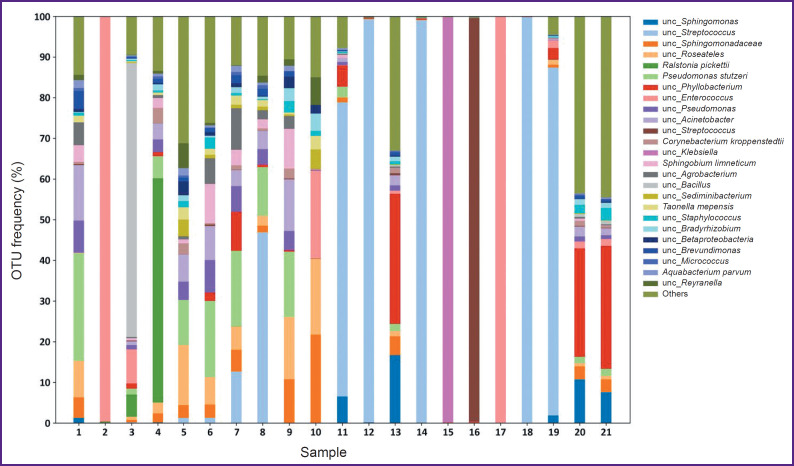
Taxonomic structure of the bacterial communities of native cardiac valves affected by infective endocarditis OTUs — operative taxonomic units

## Discussion

IE is characterized by the microorganisms, which colonize cardiac valves. Despite the advances in the disease prevention, diagnosis and therapy, currently, the fatality rate remains high: nosocomial fatality is from 6 to 50%, 5-year death rate — from 19 to 82% [20]. Epidemiological studies show IE incidence to be from 5.0 to 14.3 cases per 100,000 thousand of adult population [21]. Moreover, IE etiological structure has been found to have changed. So, at present, the main agents (*Streptococcus* spp. and *Staphylococcus* spp.) account for nearly 80% of IE cases. In approximately 10% cases the etiological agents are enterococci, particularly, *Enterococcus faecalis*, and their percentage is higher in patients aged over 65 [22]. Gram-negative rods including those from HACEK group (*Haemophilus*, *Aggregatibacter*, *Cardiobacterium*, *Eikenella*, and *Kingella*) are rarely detected (their portion is about 5% of IE cases).

Correct agent identification is a crucially important aspect of IE diagnosis [4]. A conventional definition of IE etiological agent by blood culture or incised tissue can give false-negative results in the range from 2.5 to 31.0% cases; and it is frequently associated with antibiotic therapy prior to admission [20]. In addition, a number of bacteria causing IE can refer to difficult-to-culture or non-cultured groups [23]. The present study demonstrated that the agents revealed by microbiological tests accounted for about 30% of the studied IE cases, while the identification of bacterial agents using 16S rRNA metabarcoding was 100%. So, 16S rRNA enabled to reveal the following bacteria: *Streptococcus*, *Enterococcus*, *Sphingomonas*, *Roseateles*, *Phyllobacterium*, *Pseudomonas*, *Ralstonia*, *Bacillus*, and *Klebsiella*. None *Staphylococcus* cases were found, despite the fact that these microorganisms are one of the most common agents affecting cardiac valval tissues.

*Streptococcus* representatives rank next to *Staphylococcus aureus* in IE pathogenesis. *Streptococcus* is a heterogeneous group of bacteria containing 50 identified species. Some researchers demonstrate that particular streptococci, primarily from *Mitis* group, are the main IE agents [24, 25]. *Streptococcus gordonii* and *Streptococcus mutans* were shown to be the most common streptococci (they were found in 50% cases), *Streptococcus pneumoniae* and *Streptococcus pyogenes* were rarely found (1–2%) [25]. *Enterococcus* includes gram-positive bacteria holding leading positions among IE agents. Up to 20% of all IE cases can be due to these bacteria, primarily, *Enterococcus faecalis* [26]. The therapy of IE caused by *Enterococcus* encounters certain difficulties, since the pathogen is able to form biofilms and preserve microorganisms on both: native cardiac valves, and prosthetic valval structures [27, 28]. It is worth noting that compared to other etiologies, IE caused by *E. faecalis* is characterized by recurrences after cessation of antibiotic therapy [29, 30].

Moreover, our study revealed two cases of bacterial invasion by rarer microorganisms, such as *Ralstonia pickettii* and *Bacillus* spp. *R. pickettii* belonging to *Ralstonia* is an aerobic gram-negative oxidase-positive bacillus, which is recently considered as an opportunistic pathogen in patients with compromised immunity. *R. pickettii* can survive in a wide temperature range (from 15 to 42°C), as well as it forms a biofilm resistant to a host immune response and some antibiotics [31]. In addition, currently, there is no standardized protocol to treat infections caused by *R. pickettii* due to the differences in the sensitivity to antibiotics. Immunosuppressed patients, especially those with acquired (HIV) or pharmaceutical immunosuppression are at risk of getting infected with this type of bacteria [32] that is described by our case of detecting *R. pickettii* in a patient with involved aortal valve and accompanying HIV and hepatitis C.

In one case 16S rRNA metabarcoding resulted in revealing and identifying *Bacillus* agent in the patient’s mitral valve, its blood culture showed positive *E. faecalis. Bacillus* bacteria are gram-positive facultative anaerobes occurring in the environment, food, and in the intestinal microflora [33]. Moreover, *Bacillus* bacteria have been found to be able to cause IE involving in most cases the cardiac mitral valve, and be associated with heavy mortality in the patients with prosthetic valval structures [34]. Endocarditis caused by *Bacillus* has been shown to occur frequently in patients with intravenous drug taking, venous catheters, cardiac valval prostheses, malignancies, and immunosuppression [35].

It is of importance to note that the present study revealed one case of gram-negative *Klebsiella*. The most common *Klebsiella* species are *Klebsiella pneumoniae* and *Klebsiella oxytoca*. *Klebsiella granulomatis*, *Klebsiella ozaenae*, and *Klebsiella rhinoscleromatis* occur rarer. Among the common species it is *K. pneumoniae* that frequently causes IE, and is characterized by a severe course, mainly due to the fact that these bacteria are resistant to some antibiotics [36].

## Conclusion

Thus, the present study findings showed the native cardiac valves excised in IE patients to be characterized by numerous opportunistic pathogenic microorganisms, although blood cultures were negative. It suggests that the residual bacteriemia in patients with preoperative antibacterial therapy aimed primarily at common IE agents can result in developing postoperative prosthetic IE followed by a re-operation, life quality degradation and an increased risk of mortality. The obtained results demonstrated the significance of 16S rRNA metabarcoding of the removed cardiac valves and the necessity for advanced study of taxonomic variety of cardiac valves affected by IE using high-efficiency methods.

## References

[1] Li M., Kim J.B., Sastry B.K.S., Chen M (2024). Infective endocarditis.. Lancet.

[2] Martínez-Sellés M., Muñoz P (2023). Epidemiology, diagnosis, treatment, and prognosis of infective endocarditis.. J Clin Med.

[3] Stasev A.N., Rutkovskaya N.V., Kokorin S.G., Levadin Yu.V (2017). Salmonella endocarditis of mitral valve: clinical observation.. Complex Issues of Cardiovascular Diseases.

[4] Oberbach A., Schlichting N., Feder S., Lehmann S., Kullnick Y., Buschmann T., Blumert C., Horn F., Neuhaus J., Neujahr R., Bagaev E., Hagl C., Pichlmaier M., Rodloff A.C., Gräber S., Kirsch K., Sandri M., Kumbhari V., Behzadi A., Behzadi A., Correia J.C., Mohr F.W., Friedrich M (2017). New insights into valve-related intramural and intracellular bacterial diversity in infective endocarditis.. PLoS One.

[5] Demin A.A., Kobalava Zh.D., Skopin I.I., Tyurin P.V., Boytsov S.A., Golukhova E.Z., Gordeev M.L., Gudymovich V.D., Demchenko E.A., Drobysheva V.P., Domonova E.A., Drapkina O.M., Zagorodnikova K.A., Irtyuga O.B., Kakhktsyan P.S., Kozlov R.S., Kotova E.O., Medvedev A.P., Muratov R.M., Nikolaevsky E.N., Pisaryuk A.S., Ponomareva E.Yu., Popov D.A., Rakhina S.A., Revishvili A.G., Reznik I.I., Ryzhkova D.S., Safarova A.F., Tazina S.Ya., Chipigina N.S., Shipulina O.Yu., Shlyakhto E.S., Schneider Yu.A., Shostak N.A (2022). Infectious endocarditis and infection of intracardiac devices in adults. Clinical guidelines 2021.. Russian Journal of Cardiology.

[6] Kotova E.O., Domonova E.A., Kobalava Z.D., Moiseeva A.Y., Pisaryuk A.S., Silveystrova O.Y., Karaulova J.L., Akimkin V.G (2023). Clinical and diagnostic value of including PCR blood test in the traditional algorithm for identifying causative agents of infective endocarditis: a cohort study of 124 patients.. Terapevticheskii arkhiv.

[7] Zeng X., Wu J., Li X., Xiong W., Tang L., Li X., Zhuang J., Yu R., Chen J., Jian X., Lei L (2022). Application of metagenomic next-generation sequencing in the etiological diagnosis of infective endocarditis during the perioperative period of cardiac surgery: a prospective cohort study.. Front Cardiovasc Med.

[8] Haddad S.F., DeSimone D.C., Chesdachai S., Gerberi D.J., Baddour L.M (2022). Utility of metagenomic next-generation sequencing in infective endocarditis: a systematic review.. Antibiotics (Basel).

[9] Burban A., Słupik D., Reda A., Szczerba E., Grabowski M., Kołodzińska A (2024). Novel diagnostic methods for infective endocarditis.. Int J Mol Sci.

[10] Martinez-Porchas M., Villalpando-Canchola E., Ortiz Suarez L.E., Vargas-Albores F (2017). How conserved are the conserved 16S-rRNA regions?. PeerJ.

[11] Anton-Vazquez V., Dworakowski R., Cannata A., Amin-Youssef G., Gunning M., Papachristidis A., MacCarthy P., Baghai M., Deshpande R., Khan H., Byrne J., Fife A (2022). 16S rDNA PCR for the aetiological diagnosis of culture-negative infective endocarditis.. Infection.

[12] Fadrosh D.W., Ma B., Gajer P., Sengamalay N., Ott S., Brotman R.M., Ravel J (2014). An improved dual-indexing approach for multiplexed 16S rRNA gene sequencing on the Illumina MiSeq platform.. Microbiome.

[13] Shen W., Le S., Li Y., Hu F (2016). SeqKit: a cross-platform and ultrafast toolkit for FASTA/Q file manipulation.. PLoS One.

[14] https://www.bioinformatics.babraham.ac.uk/projects/fastqc/.

[15] Edgar R.C (2013). UPARSE: highly accurate OTU sequences from microbial amplicon reads.. Nat Methods.

[16] Edgar R.C (2010). Search and clustering orders of magnitude faster than BLAST.. Bioinformatics.

[17] Edgar R.C (2016). UNOISE2: improved error-correction for Illumina 16S and ITS amplicon sequencing.. bioRxiv.

[18] Edgar R.C (2016). SINTAX: a simple non-Bayesian taxonomy classifier for 16S and ITS sequences.. bioRxiv.

[19] Wang Q., Garrity G.M., Tiedje J.M., Cole J.R (2007). Naive Bayesian classifier for rapid assignment of rRNA sequences into the new bacterial taxonomy.. Appl Environ Microbiol.

[20] Kouijzer J.J.P., Noordermeer D.J., van Leeuwen W.J., Verkaik N.J., Lattwein K.R (2022). Native valve, prosthetic valve, and cardiac device-related infective endocarditis: a review and update on current innovative diagnostic and therapeutic strategies.. Front Cell Dev Biol.

[21] Thornhill M.H., Dayer M.J., Nicholl J., Prendergast B.D., Lockhart P.B., Baddour L.M (2020). An alarming rise in incidence of infective endocarditis in England since 2009: why?. Lancet.

[22] Kobalava Z.D., Kotova E.O (2023). Global and national trends in the evolution of infective endocarditis.. Kardiologiia.

[23] Reisinger M., Kachel M., George I (2024). Emerging and re-emerging pathogens in valvular infective endocarditis: a review.. Pathogens.

[24] Cai S., Yang Y., Pan J., Miao Q., Jin W., Ma Y., Zhou C., Gao X., Wang C., Hu B (2021). The clinical value of valve metagenomic next-generation sequencing when applied to the etiological diagnosis of infective endocarditis.. Ann Transl Med.

[25] Kim S.L., Gordon S.M., Shrestha N.K (2018). Distribution of streptococcal groups causing infective endocarditis: a descriptive study.. Diagn Microbiol Infect Dis.

[26] Chamat-Hedemand S., Dahl A., Østergaard L., Arpi M., Fosbøl E., Boel J., Oestergaard L.B., Lauridsen T.K., Gislason G., Torp-Pedersen C., Bruun N.E (2020). Prevalence of infective endocarditis in streptococcal bloodstream infections is dependent on streptococcal species.. Circulation.

[27] Habib G., Lancellotti P., Erba P.A., Sadeghpour A., Meshaal M., Sambola A., Furnaz S., Citro R., Ternacle J, Donal E., Cosyns B., Popescu B., Iung B., Prendergast B., Laroche C., Tornos P., Pazdernik M., Maggioni A., Gale C.P., EURO-ENDO Investigators (2019). The ESC-EORP EURO-ENDO (European Infective Endocarditis) registry.. Eur Heart J Qual Care Clin Outcomes.

[28] Conwell M., Dooley J.S.G., Naughton P.J (2022). Enterococcal biofilm — a nidus for antibiotic resistance transfer?. J Appl Microbiol.

[29] Lecomte R., Laine J.B., Issa N., Revest M., Gaborit B., Le Turnier P., Deschanvres C., Benezit F., Asseray N., Le Tourneau T., Pattier S., Al Habash O., Raffi F., Boutoille D., Camou F (2020). Long-term outcome of patients with nonoperated prosthetic valve infective endocarditis: is relapse the main issue?. Clin Infect Dis.

[30] Calderón-Parra J., Kestler M., Ramos-Martínez A., Bouza E., Valerio M., de Alarcón A., Luque R., Goenaga M.Á., Echeverría T., Fariñas M.C., Pericàs J.M., Ojeda-Burgos G., Fernández-Cruz A., Plata A., Vinuesa D., Muñoz P., on behalf of the GAMES investigators (2021). Clinical factors associated with reinfection versus relapse in infective endocarditis: prospective cohort study.. J Clin Med.

[31] Danneels P., Hamel J.F., Picard L., Rezig S., Martinet P., Lorleac’h A., Talarmin J.P., Buzelé R., Guimard T., Le Moal G., Brochard-Libois J., Beaudron A., Letheulle J., Codde C., Chenouard R., Boutoille D., Lemaignen A., Bernard L., Cattoir V., Dubée V, EFEMER study group (2023). Impact of Enterococcus faecalis endocarditis treatment on risk of relapse.. Clin Infect Dis.

[32] Basso M., Venditti C., Raponi G., Navazio A.S., Alessandri F., Giombini E., Nisii C., Di Caro A., Venditti M (2019). A case of persistent bacteraemia by Ralstonia mannitolilytica and Ralstonia pickettii in an intensive care unit.. Infect Drug Resist.

[33] Orme J., Rivera-Bonilla T., Loli A., Blattman N.N (2015). Native valve endocarditis due to Ralstonia pickettii: a case report and literature review.. Case Rep Infect Dis.

[34] Wright W.F (2016). Central venous access device-related bacillus cereus endocarditis: a case report and review of the literature.. Clin Med Res.

[35] Gopinathan A., Kumar A., Sen A.C., Sudha S., Varma P., Gs S., Eapen M., Dinesh K.R (2018). A case series and review of bacillus cereus endocarditis from India.. Open Microbiol J.

[36] Ioannou P., Miliara E., Baliou S., Kofteridis D.P (2021). Infective endocarditis by Klebsiella species: a systematic review.. J Chemother.

